# Fabrication of Reproducible and Selective Ammonia Vapor Sensor-Pellet of Polypyrrole/Cerium Oxide Nanocomposite for Prompt Detection at Room Temperature

**DOI:** 10.3390/polym13111829

**Published:** 2021-05-31

**Authors:** Ahmad Husain, Salma Ahmed Al-Zahrani, Ahmed Al Otaibi, Imran Khan, Mohammad Mujahid Ali Khan, Abeer Mohamed Alosaimi, Anish Khan, Mahmoud Ali Hussein, Abdullah M. Asiri, Mohammad Jawaid

**Affiliations:** 1 Department of Applied Chemistry, Faculty of Engineering and Technology, Aligarh Muslim University, Aligarh 202002, India; 2 Chemistry Department, Faculty of Science, University of Ha’il, P.O. Box 2440, Ha’il 81451, Saudi Arabia; s.alzahrane@uoh.edu.sa (S.A.A.-Z.); ahmed.alotaibi@uoh.edu.sa (A.A.O.); 3 Applied Science and Humanities Section, Faculty of Engineering and Technology, University Polytechnic, Aligarh Muslim University, Aligarh 202002, India; imrannano@gmail.com (I.K.); mujahidchemistry@gmail.com (M.M.A.K.); 4 Department of Chemistry, Faculty of Science, Taif University, P.O. Box 11099, Taif 21944, Saudi Arabia; abeer_alosaim@hotmail.com; 5 Chemistry Department, Faculty of Science, King Abdulaziz University, P.O. Box 80203, Jeddah 21589, Saudi Arabia; mahussein73@yahoo.com (M.A.H.); asiri2@kau.edu.sa (A.M.A.); 6 Center of Excellence for Advanced Materials Research, King Abdulaziz University, P.O. Box 80203, Jeddah 21589, Saudi Arabia; 7 Chemistry Department, Faculty of Science, Assiut University, Assiut 71516, Egypt; 8 Laboratory of Biocomposite Technology, Institute of Tropical Forestry and Forest Products (INTROP), University Putra Malaysia, UPM Serdang, Selangor 43400, Malaysia; jawaid@upm.edu.my

**Keywords:** polypyrrole, cerium oxide, nanocomposite, DC electrical conductivity, ammonia vapor sensing

## Abstract

Polypyrrole (PPy) and polypyrrole/cerium oxide nanocomposite (PPy/CeO_2_) were prepared by the chemical oxidative method in an aqueous medium using anhydrous ferric chloride (FeCl_3_) as an oxidant. The successful formulation of materials was confirmed by Fourier transform infrared spectroscopy (FT-IR), X-ray diffraction (XRD), thermogravimetric analysis (TGA), scanning electron microscopy (SEM), and transmittance electron microscopy (TEM). A four-in-line probe device was used for studying DC electrical conductivity and ammonia vapor sensing properties of PPy and PPy/CeO_2_. The significant improvement in both the conductivity and sensing parameters of PPy/CeO_2_ compared to pristine PPy reveals some synergistic/electronic interaction between PPy and cerium oxide nanoparticles (CeO_2_ NPs) working at molecular levels. The initial conductivity (i.e., conductivity at room temperature) was found to be 0.152 Scm^−1^ and 1.295 Scm^−1^ for PPy and PPy/CeO_2_, respectively. Also, PPy/CeO_2_ showed much better conductivity retention than pristine PPy under both the isothermal and cyclic ageing conditions. Ammonia vapor sensing was carried out at different concentration (0.01, 0.03, 0.05, 0.1, 0.2, 0.3, 0.4, and 0.5 vol %). The sensing response of PPy/CeO_2_ varied with varying concentrations. At 0.5 vol % ammonia concentration, the % sensing response of PPy and PPy/CeO_2_ sensor was found to be 39.1% and 93.4%, respectively. The sensing efficiency of the PPy/CeO_2_ sensor was also evaluated at 0.4. 0.3, 0.2, 0.1, 0.05, 0.03, and 0.01 vol % ammonia concentration in terms of % sensing response, response/recovery time, reversibility, selectivity as well as stability at room temperature.

## 1. Introduction

Recently, due to rapid industrial development and uncontrolled human activities, the release of toxic gases into the environment has increased many folds. Ammonia is one of the most harmful environmental pollutants. If the concentration of ammonia in the environment exceeds 300 ppm, it may damage the human cell [1,2,3,4,5]. It may also cause many diseases of the eyes, kidneys, liver, respiratory tract, as well as skin. Hence, for the sake of our ecosystem and good human health, the detection and monitoring of highly toxic ammonia at room temperature is a necessity [1,2,3,4,5]. In the past few years, conducting polymers have been used for the fabrication of highly efficient chemiresistive ammonia sensor working at ambient temperature. Lately, a great deal of research was carried out in the arena of conducting polymers as they possess good electrical properties and find application in various fields of science and technology, especially in gas/vapor sensing devices [1,2,3,4,5,6,7,8,9,10,11,12,13,14,15,16,17,18,19,20].

Amongst the several conducting polymers present today, polypyrrole (PPy) has garnered great interest because of its high conductivity, high environmental and thermal stability, low toxicity, and facile synthesis. The important thing about PPy based sensors is that they can be operated at room temperature. The electrical and morphological properties of PPy can easily be tuned by controlling both the polymerization method and doping process. The sensors based on PPy showed quick response at room temperature even at very low concentration of analyte gases/vapors. The main drawback of these sensors is poor selectivity and long-term stability [1,2,3,4,5,6]. Both the sensing performance and electrical conductivity of pristine PPy could be enhanced significantly by the formulation of its nanocomposites. Over the years, different types of nano-filler—such as metal/semi-metal oxides, graphene, carbon nanotubes, etc.—have been incorporated into the PPy matrix. The improved properties of nanocomposites are explained by the electronic/synergistic interaction between PPy chains and nano-filler acting at molecular levels [15,16,17,21,22,23,24,25].

Among them, metal oxides are cheap, environmentally stable and can be easily obtained [26,27]. Cerium oxide (CeO_2_) is abundant as well as an attractive rare earth oxide which is also the cheapest electrode materials amongst the existing metal oxides today. It finds application in various fields such as preventing corrosion, thermal coatings, electromagnetic shielding, as well in electrochemical cells [28,29,30,31]. It exists in two oxidation states—i.e., +3 and +4, respectively—although most rare earth metals exist only in the +3 oxidation state. As it possesses the ability to transform its oxidation state, it is used in a variety of applications such as solid oxide fuel cells as well as catalytic converters [32,33]. Also, CeO_2_ has attracted great attention as it possesses optical and thermal properties and unique characteristics such as being non-toxic, biocompatible, and having the ability to store oxygen [34]. There are several studies reporting the formulation of composites of PPy with CeO_2_ and its utilization in chemical and bio-sensing devices.

Khan et al. fabricated a highly sensitive and sophisticated sensor of PPy/CeO_2_/glassy carbon electrode for electrochemically detecting flupirtine maleate [35]. Karimi et al. synthesized an H_2_O_2_ electrochemiluminescence (ECL) sensor by polypyrrole-dodecylbenzene sulfate-cerium oxide nanocomposite on a Pt electrode which possessed a very low detection limit [36]. The effect of CeO_2_ on the structure, morphology, as well as dielectric properties of PVA/PPy blend composites, was studied by Mohanpriya et al. [37]. The DC conductivity as well as sensing behavior of nanostructured polypyrrole-CeO_2_ composites towards LPG was studied by Seema et al. [38]. The effect CeO_2_ incorporation has on the ion-exchange properties of polypyrrole films doped by dodecylsulfate was investigated by Benmouhoub et al. [39].

To our best knowledge, PPy/CeO_2_ nanocomposite is not utilized for ammonia vapor sensing, yet. Hence, motivated by exceptional results of PPy/CeO_2_ based sensors towards a large number of the analyte, we have prepared PPy and PPy/CeO_2_ through chemical oxidative polymerization. The prepared materials were characterized by several advanced techniques. DC electrical conductivity retention ability of PPy and PPy/CeO_2_ was also tested under isothermal and cyclic ageing conditions. Ammonia vapor sensing properties of PPy and PPy/CeO_2_ based sensor-pellets at room temperature were evaluated and compared. The sensing efficiency of the PPy/CeO_2_ sensor was evaluated at different ammonia concentrations *viz.* 0.01, 0.03, 0.05, 0.1, 0.2, 0.3, 0.4, and 0.5 vol % with respect to % sensing response, response/recovery time, reproducibility, selectivity and stability.

To our best knowledge, PPy/CeO_2_ nanocomposite is utilized first time as the pellet-shaped sensor for fast, reversible and selective detection of ammonia vapor at room temperature.

## 2. Experimental

### 2.1. Materials

Pyrrole 99% (Sigma-Aldrich, Aligarh, India), anhydrous ferric chloride (FeCl_3_) and methanol (Fischer Scientific, Aligarh, India), cerium oxide nanoparticles (CeO_2_ NPs) having particle size 20–50 nm from Platonic Nanotech Pvt. Ltd., Jharkhand, India were used as received. Double-distilled water was used in synthesis and other experiments.

### 2.2. Synthesis of PPy and PPy/CeO_2_

PPy and PPy/CeO_2_ were synthesized by in-situ oxidative polymerization method in an aqueous medium using FeCl_3_ as an oxidant. In the first step of the synthesis, in 100 mL of double-distilled water, 0.03 mol of pyrrole was agitated. Then an aqueous solution of FeCl_3_ (0.09 mol) in 100 mL double-distilled water was prepared. Subsequently, FeCl_3_ solution was added dropwise to the aqueous suspension of pyrrole during continuous stirring condition at room temperature. The stirring was continued for 10 h that led to the formation of a black slurry. This step was followed by simultaneous filtration and washing of thus obtained black colored slurry with double-distilled water and methanol. Thus, obtained black colored PPy was dried in an air oven at 60 °C for 12 h, converted to a fine powder and was kept in a desiccator for carrying out further investigations.

**Figure 1:**
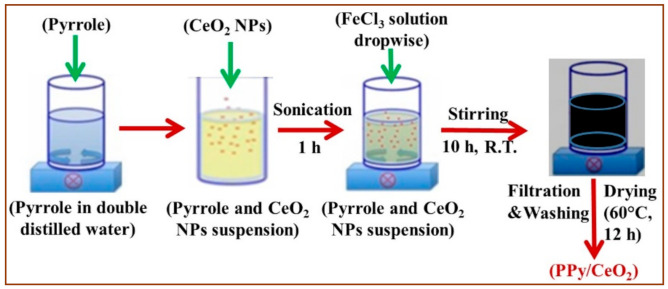
Schematic diagram showing various steps involved in the synthesis of PPy/CeO_2_.

An identical procedure was adopted to synthesize the PPy/CeO_2_. First of all, 0.03 mol of pyrrole was agitated in 100 mL of double-distilled water. Then, 500 mg of CeO_2_ NPs were transferred into the above aqueous suspension of pyrrole. Then, pyrrole and CeO_2_ NPs suspension were ultrasonicated for 1 h intended for the adsorption of pyrrole molecules on the surface of CeO_2_ NPs. After that, FeCl_3_ solution was added dropwise to the aqueous suspension of pyrrole and CeO_2_ NPs during continuous stirring condition at room temperature for 10 h, which produced a black colored slurry of PPy/CeO_2_. Thus, prepared slurry of PPy/CeO_2_ was filtered with continuous washing with double-distilled water and methanol. Thus, produced black colored PPy/CeO_2_ was dried in an air oven at 60 °C for 12 h, converted to a fine powder and was kept in a desiccator for carrying out further investigations (Figure 1).

### 2.3. Characterization and Instrumentation

In order to study the crystalline structure and phase of PPy, CeO_2_ NPs, and PPy/CeO_2,_ an X-ray beam diffractometer (Shimadzu XRD, model 6100, Kyoto, Japan) (Cu Kα radiation (1.540 Å)) operating at a voltage of 30 kv and 2θ value between 5 and 80° was employed. In order to record the Fourier transform infrared (FT-IR) spectra of PPy, CeO_2_ NPs and PPy/CeO_2_ in the range of 400−4000 cm^−1^ a Perkin-Elmer-1725 (Waltham, MA, USA) on KBr pellets was used. The surface morphological studies of PPy and PPy/CeO_2_ were performed using a JSM-6510-LV (JEOL, Tokyo, Japan) scanning electron microscope and JEM-2100 (JEOL, Tokyo, Japan) transmission electron microscope, respectively. In order to carry out the thermogravimetric analysis a Shimadzu 60 H instrument (Kyoto, Japan) was used where the samples were heated in the range 50 °C to 900 °C at a rate of 15 °C min^−1^ in an atmosphere of nitrogen with a flow rate of 150 mL/min.

DC electrical conductivity and ammonia vapor sensing experiments were done by a four-in-line probe instrument attached with a PID controlled oven manufactured by Scientific Equipment, Roorkee, India. The equation used for the calculation of electrical conductivity is

σ = [ln2 (2S/W)]/[2πS (V/I)](1)

where: I, V, W, S, and σ represent the current (A), voltage (V), the thickness of the pellet (cm) probe spacing (cm) and conductivity (Scm^−1^), respectively [40,41,42].

The pellets of PPy and PPy/CeO_2_ for conductivity and sensing studies were made by a hydraulic pressure machine at 80 kN pressure applied for 120 s. 200 mg of each sample was used for the preparation of Pellets. Different pellets were used in conductivity and sensing experiments.

In isothermal ageing experiments, the pellet of PPy and PPy/CeO_2_ were heated at 50, 70, 90, 110, and 130 °C in PID controlled air oven. The electrical conductivity was calculated at a particular temperature at an interval of 10 min. In cyclic ageing experiments, the conductivity was determined for four successive cycles starting from 50 °C to 130 °C [43,44].

## 3. Results and Discussion

### 3.1. Fourier Transform Infrared Spectroscopic (FT-IR) Studies

**Figure 2:**
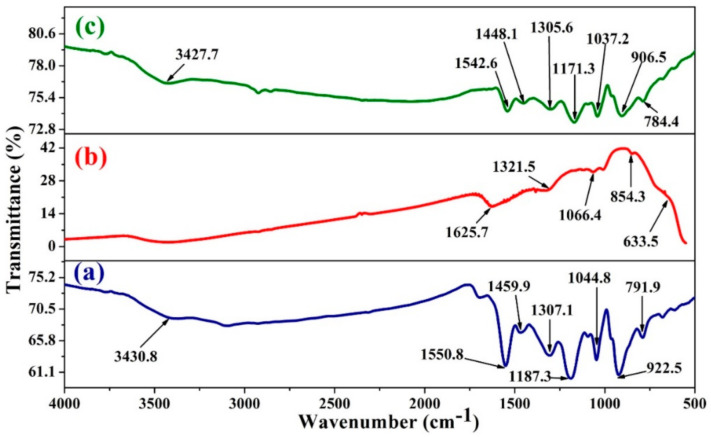
FTIR spectra of: (**a**) PPy, (**b**) CeO_2_ NPs, and (**c**) PPy/CeO_2_.

The IR spectra of PPy, CeO_2_ NPs, and PPy/CeO_2_ are shown in Figure 2. In PPy spectrum (Figure 2a), the peak at 3430.8 cm^−1^ is owing to the N-H stretching vibrations. The peak at 1550.8 cm^−1^ is a result of C=C stretching vibrations. The peak at 1459.9 cm^−1^ corresponds to the C-C stretching and the peaks appeared 1307.1 cm^−1^ and 1187.3 cm^−1^ may be due to the C=N bending and C-N vibration, respectively. The =C-H bending is shown by the peak at 1044.8 cm^−1^, while the peak at 791.9 cm^−1^ is attributed to the vibrations of C-H bonds. The peak at 922.5 cm^−1^ is ascribed to the =C-N^+^-C stretching vibration. This peak at 922.5 cm^−1^ shows that PPy was effectively oxidized and doped by FeCl_3_ into positively charged species that act as charge carriers (i.e., polarons/bipolarons) [17,23,24].

In the spectrum of CeO_2_ NPs (Figure 2b), peak is observed at 1625.7 cm^−1^ because of the O-H stretching vibration of the water molecule. The peaks at 1321.5 cm^−1^ and 1066.4 cm^−1^ can be related to the carbonate-like species formation on the surface of CeO_2_ NPs. The peak at 854.3 cm^−1^ is because of enveloping of the phonon band of CeO_2_ NPs. The broad peak at 633.5 cm^−1^ is due to the vibrational mode of O-Ce-O bonds [45,46,47].

In the spectrum of PPy/CeO_2_ (Figure 2c), all the characteristic peaks of PPy were weakened and observed at smaller wavenumbers (Table 1). The weakening and shifting of peaks may be due to some synergistic/electronic interaction between PPy chains and molecular interacting CeO_2_ NPs working at molecular levels. There was no visible peak of CeO_2_ NPs in PPy/CeO_2_ spectrum which showed encapsulation of CeO_2_ NPs into PPy matrix (can be confirmed by SEM and TEM images).

### 3.2. X-ray Diffraction (XRD) Studies

**Figure 3:**
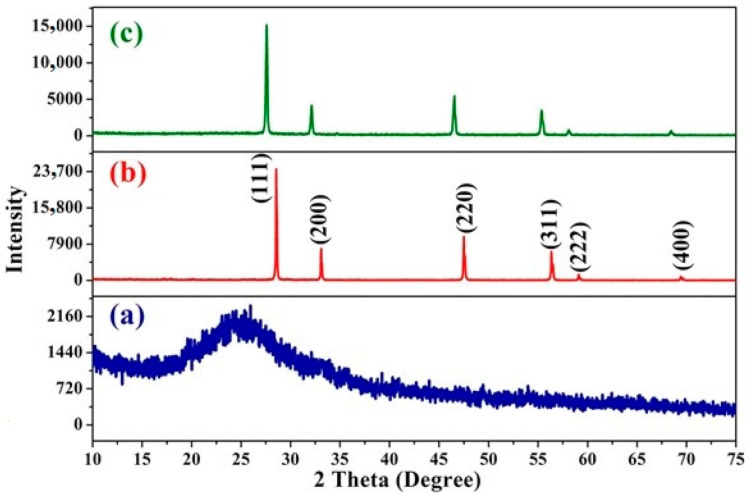
XRD spectra of: (**a**) PPy, (**b**) CeO_2_ NPs and (**c**) PPy/CeO_2_.

The XRD spectra of PPy, CeO_2_ NPs and PPy/CeO_2_ are displayed in Figure 3. In PPy (Figure 3a), the broad band between 2θ = 20–30°, having a maximum at 2θ = 24.87° showed pyrrole was successfully polymerized to PPy. The broad also confirms that PPy was amorphous in nature [17,23,24].

The XRD spectrum of CeO_2_ NPs (Figure 3b) consists of several peaks at 2θ = 28.57°, 33.14°, 47.59°, 56.43°, 59.1°, and 69.56° which are related to the crystalline planes (111), (200), (220), (311) (222), and (400), respectively [45,46,47].

In PPy/CeO_2_ (Figure 3c), all the characteristic crystalline planes of CeO_2_ NPs were observed to confirm the presence of CeO_2_ NPs in PPy/CeO_2_. The peaks of CeO_2_ NPs were observed at 2θ = 27.54°, 32.17°, 46.52°, 55.38°, 58.16°, and 68.50° respectively. The most significant observation was the shifting of peaks of CeO_2_ NPs towards lower 2θ angles along with a decrease in intensity. The observed peak shifting along with a decrease in intensity can be confirmation of the thorough incorporation of CeO_2_ NPs into the PPy matrix.

### 3.3. Morphological Studies (SEM and TEM)

**Figure 4:**
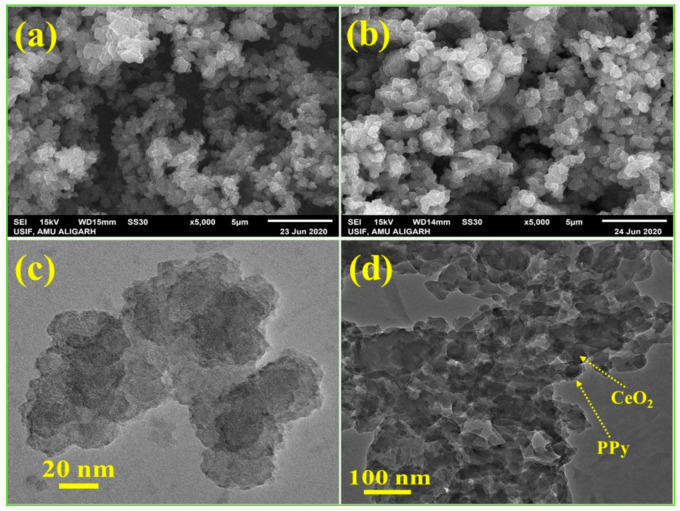
SEM and TEM images of PPy (**a**,**c**) and PPy/CeO_2_ (**b**,**d**), respectively.

The scanning electron micrographs and transmittance electron micrographs of PPy and PPy/CeO_2_ are shown in Figure 4. From the SEM and TEM images of PPy (Figure 4a,c, respectively), it is evident that the morphology of pristine PPy consists of several globular particles which have been agglomerated [17,23,24]. In the case of PPy/CeO_2_ (Figure 4b), a similar morphology as pristine PPy is observed consisting of clusters of globular particles. The most important point is that there was no distinct CeO_2_ NPs in the SEM image of PPy/CeO_2_ indicating the successful polymerization of pyrrole on the surface of CeO_2_ NPs. The presence of CeO_2_ NPs into the matrix of PPy can be seen in the TEM image of PPy/CeO_2_ (Figure 4d). The homogeneous and complete incorporation of CeO_2_ NPs into the PPy matrix confirmed the successful formulation of PPy/CeO_2_ nanocomposite.

### 3.4. Thermogravimetric Analysis (TGA)

**Figure 5:**
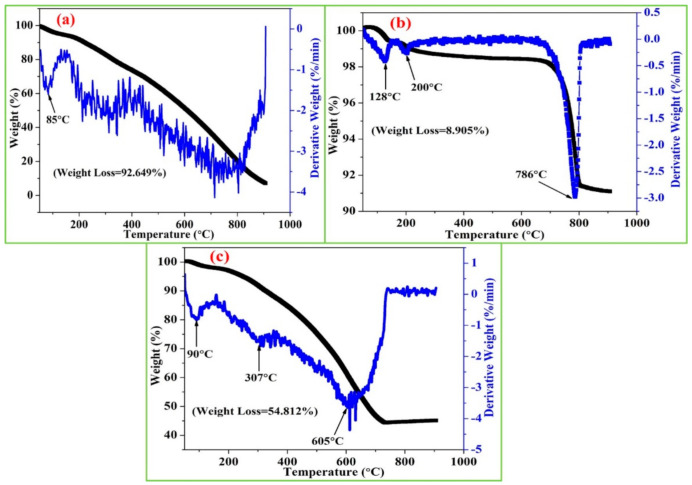
TGA curve of (**a**) PPy, (**b**) CeO_2_ NPs, and (**c**) PPy/CeO_2_.

The TGA curve of PPy, CeO_2_ NPs, and PPy/CeO_2_ are displayed in Figure 5. In the case of PPy (Figure 5a), the first weight loss at 85 °C is due to the loss of water in form of moisture. The observed subsequent regular weight loss at higher temperatures is related to dopant loss and degradation of the PPy matrix [23,24]. The total weight loss was found to be 92.649%.

In the case of CeO_2_ NPs (Figure 5b), the total weight loss was found to be only 8.905% which shows outstanding stability. Thus, the incorporation of CeO_2_ NPs into the PPy matrix proved to be very effective for increasing the thermal stability of PPy/CeO_2_ as evident from Figure 5c. For PPy/CeO_2_, weight loss at 90, 307, and 605 °C is caused by the loss of water, removal of dopant and degradation of PPy matrix. The total weight loss was found to be 54.812% which is very less as compared to pristine PPy (92.649%). Thus, extra stability of PPy/CeO_2_ also confirms the formulation of nanocomposite due to some interaction between PPy chains and CeO_2_ NPs at molecular levels. The greater conductivity retention of PPy/CeO_2_ than pristine PPy is due to greater thermal stability. This facilitates PPy/CeO_2_ as the potential material for electrical conductor applications working above room temperature.

### 3.5. DC Electrical Conductivity Studies

**Figure 6:**
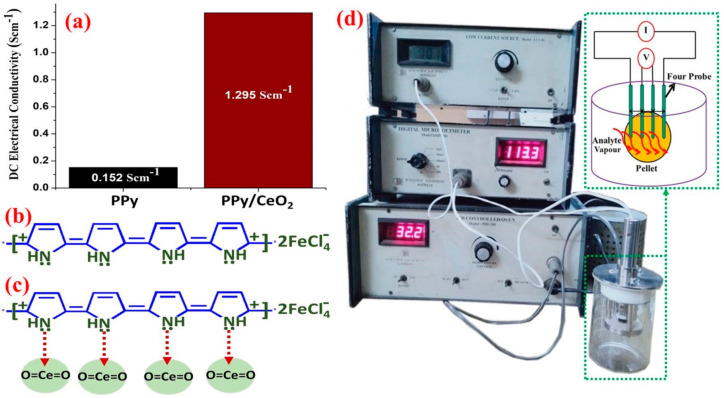
Initial conductivity of: (**a**) PPy and PPy/CeO_2_, (**b**) charge carriers of PPy generated due to oxidation/doping, (**c**) the interaction of PPy chain with CeO_2_ NPs leading to the formation of more efficient electronic paths needed for conductivity enhancement, and (**d**) instrumental setup used in conductivity and sensing experiments.

Initial electrical conductivity (at 29 °C and 44% humidity) of PPy and PPy/CeO_2_ was found to be 0.152 Scm^−1^ and 1.295 Scm^−1^, respectively (Figure 6a). The combination of CeO_2_ NPs with PPy matrix increased the conductivity about 8.5 times than pristine PPy. The charge carriers of PPy and PPy/CeO_2_ along with possible interaction of PPy chains with CeO_2_ NPs are presented in Figure 6b, c, respectively. In the case of conducting polymer, the charge carriers (polarons and bipolarons) are very similar to holes as in semiconducting materials. The electrical conductivity is explained by both the quantity and mobility of these charge carriers. Thus, the electrical conductivity can be controlled and tuned by any interaction that can change the quantity and mobility of charge carriers. Consequently, the electrical conductivity can precisely be tuned by changing both the type and level of oxidation as well as the types and amount of filler components [23,24,40,41,42,43,44]. In conducting polymer-nanocomposites, the mechanism of conductivity is described by the movement of charge carriers along with the extended π-conjugated system of polymer backbone or/and the hopping/tunnelling between fillers and polymer chains [23,43,48,49]. Pristine conducting polymers act as good semiconductor at room temperature. However, at the higher temperature, their conductivity decreases even sometimes completely lost due to degradation of the polymer backbone. However, their nanocomposites act as good semiconductor/conductor even at elevated temperatures due to greater thermal stability imparted by nanofillers, better polymer chain alignment and enhancement in the π-conjugated system provided by synergistic/electronic interaction between polymer chains and filler inorganic nanoparticles at molecular levels [48,49].

Herein, we explained significant improvement in the conductivity of PPy/CeO_2_ as compared to pristine PPy as follows: (1) The CeO_2_ NPs make available a large surface area for the polymerization of pyrrole, thus generating a more efficient and extended π-conjugated system for charge carries transportation as compared to pristine PPy. (2) The possible electronic interaction of lone pairs of nitrogen atoms of PPy with cerium (Ce^+4^) ions as well as highly electronegative oxygen atoms of CeO_2_ NPs may increase the number of charge carriers. (3) Reduction in hopping/tunnelling distance between metallic regions and improved density of charge carriers.

#### 3.5.1. DC Electrical Conductivity Retention under Isothermal Ageing Condition

The thermal stability of PPy and PPy/CeO_2_ was determined by the conductivity retention under isothermal ageing conditions. We used the following equation to calculate relative conductivity (σ*_r_*_,*t*_) at a particular temperature

(2) $$\sigma_{r,t}=\frac{\sigma_{t}}{\sigma_{0}}$$

where σ*_t_* and σ_0_ denote the DC electrical conductivity (Scm^−1^) at time t and zero, respectively [43,44].

**Figure 7:**
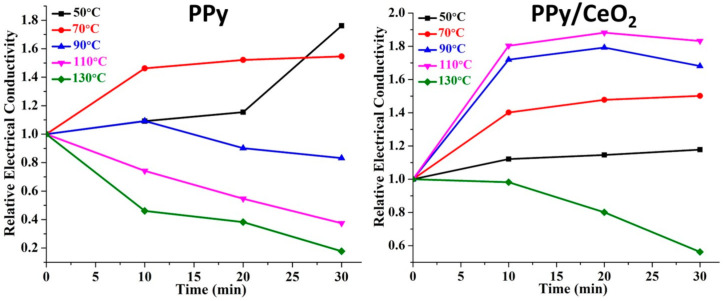
Relative electrical conductivity of PPy and PPy/CeO_2_ with respect to time.

The pristine PPy showed good conductivity retention ability at 50 °C and 70 °C. At 90 °C, the conductivity was stable upto 10 min only, then it started to decrease slightly. However, at higher temperatures (110 °C and 130 °C), the conductivity continuously decreased with respect to time which may be due to loss of moisture, dopant as well as the collapse of the π-conjugated system of the PPy chain (Figure 7) [23,24,48,49].

However, PPy/CeO_2_ displayed much-improved conductivity retention ability as compared to PPy due to greater thermal stability (Figure 7). The conductivity of PPy/CeO_2_ increased with increasing temperature showing good semiconducting nature at 50, 70, 90, and 110 °C. Also, PPy/CeO_2_ excellently retained its conductivity at 50 °C and 70 °C. At 90 °C and 110 °C, the conductivity was retained till 20 min, after that slight decrease in conductivity was observed with respect to time. At 130 °C, a sudden decrease in conductivity was detected be owing to loss of dopant plus degradation of PPy matrix.

#### 3.5.2. DC Electrical Conductivity Retention under Cyclic Ageing Condition

The thermal stability of PPy and PPy/CeO_2_ in terms of conductivity retention was also investigated under cyclic ageing conditions. The following equation was used for calculation of relative electrical conductivity (σ_r_)

(3) $$\sigma =\frac{\sigma_{T}}{\sigma_{50}}$$

where σ_T_ and σ_50_ correspond to the DC electrical conductivity at temperature T (°C) and 50 °C, respectively [43,44].

**Figure 8:**
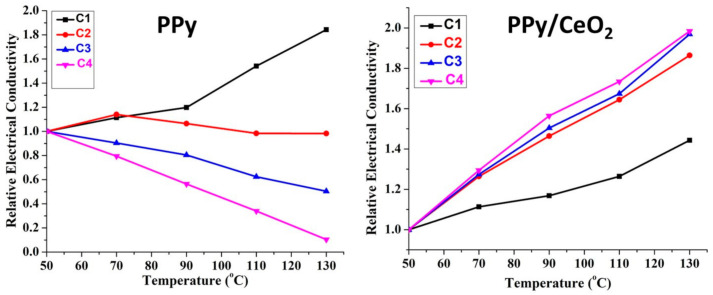
Relative electrical conductivity of PPy and PPy/CeO_2_ at different temperatures.

PPy exhibited good semiconducting properties, i.e., increase in conductivity with increasing temperature for only one cycles (Figure 8). In the second cycle, conductivity increased only up to 70 °C; after that, a slight decline was observed. For the third and fourth cycle, conductivity decreased continuously with increasing temperature due to loss of dopant as well as degradation of PPy [23,24]. However, PPy/CeO_2_ showed better stability as compared to pristine PPy. PPy/CeO_2_ was found to be stable in all four cycles. PPy/CeO_2_ showed the gain in conductivity for all four cycles which may be due to greater mobility of charge carriers at elevated temperatures (Figure 8). However, gain in conductivity in the first cycle was not as much as all three following cycles. Most importantly, for the second, third, and fourth cycles, the gain in conductivity showed an almost similar trend showing good conductivity retention ability of PPy/CeO_2_.

Thus, PPy/CeO_2_ displayed significantly improved electrical properties as compared to pristine PPy in terms of initial conductivity and conductivity retention under isothermal as well as cyclic ageing condition.

### 3.6. Ammonia Vapor Sensing Studies

**Figure 9:**
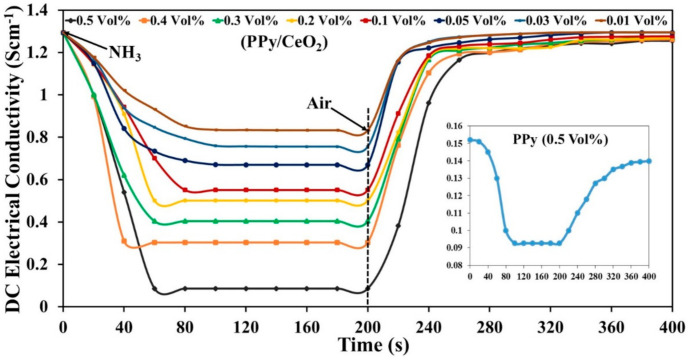
Change in conductivity of PPy/CeO_2_ and PPy (inset) sensors in ammonia vapor and air with respect to time.

Ammonia vapor sensing characteristics PPy and PPy/CeO_2_ sensor pellets were evaluated in terms of various parameters, for example, % sensing response, response time, recovery time, reversibility, selectivity, and stability at room temperature. In the end, a sensing mechanism was also proposed. To determine the effect of ammonia vapors on the electrical conductivity of sensors, first of all, the selected sensor pellet was firmly connected with the probe of the device. Then, it was kept in a closed chamber containing ammonia solutions of known concentration (Figure 6d). The pellet was exposed in ammonia vapor for 200 s for the adsorption of ammonia molecules on the sensor surface and variation in the conductivity was recorded. After that, the sensor pellet was removed from the chamber and kept in the ambient air for 200 s. A sudden and regular decrease in the sensor’s conductivity was detected in ammonia vapor, which became constant after some time. Whereas in the air, the conductivity of the sensor started to increase and became saturated (Figure 9). The % sensing response of PPy and PPy/CeO_2_ based sensors was evaluated by employing the formula

(4) $$S=\frac{\Delta \sigma }{\sigma_{i}}\times 100$$

where σ_i_ and ∆σ correspond to the initial DC electrical conductivity and change in the conductivity of sensor-pellets in the ammonia vapor for 200 s, respectively [21,40,42].

#### 3.6.1. Sensing Response

The % sensing response of PPy and PPy/CeO_2_ sensors towards ammonia vapor was calculated at 0.5 vol % concentration of ammonia solution and found to be 39.1% and 93.4%, respectively. Herein, the response time and recovery times are defined as the times required to reach 90% of the total conductivity change.

**Figure 10:**
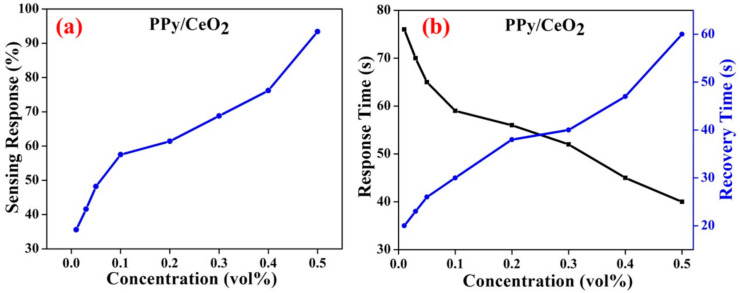
(**a**) Variation in % sensing response and (**b**) response and recovery time of PPy/CeO_2_ sensor with respect to the different ammonia concentration.

The response and recovery time for the PPy sensor at 0.5 vol % ammonia concentration was found to be about 80 s and 150 s, respectively. However, response and recovery time for PPy/CeO_2_ based sensor 0.5 vol % ammonia concentration was found to be about 40 s and 60 s, respectively. Thus, the PPy/CeO_2_ based sensor showed a much greater % sensing response and shorter response/recovery time as compared to the sensor based on pristine PPy. Therefore, % sensing response and response/recovery time PPy/CeO_2_ sensor was also calculated at 0.4, 0.3, 0.2, 0.1, 0.05, 0.03, and 0.01 vol % ammonia concentrations and found to be 76.2%, 68.8%, 61.4%, 57.5%, 48.2%, 41.6%, and 35.6%, respectively (Figure 10a). The response and recovery time was also affected significantly as ammonia concentration changes. The response/recovery time was found to be 45 s/47 s, 52 s/40 s, 56 s/38 s, 59 s/30 s, 65 s/26 s, 69 s/23 s, and 75 s/20 s at 0.4, 0.3, 0.2, 0.1, 0.05, 0.03, and 0.01 vol % ammonia concentrations, respectively (Figure 10b).

#### 3.6.2. Reversibility

**Figure 11:**
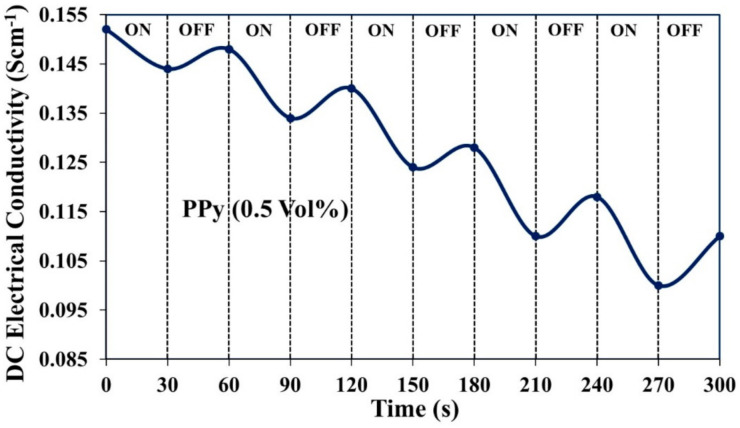
Reversibility of PPy based sensor at 0.5 vol % ammonia concentrations.

**Figure 12:**
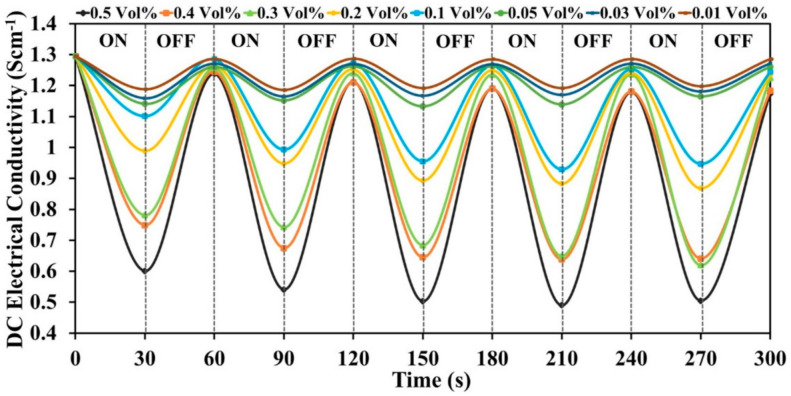
Reversibility of PPy/CeO_2_ based sensor at various ammonia concentrations.

To determine the reproducibility of the sensor, the selected sensor pellet was exposed alternately in ammonia vapor for the 30 s and air for 30 s to complete one cycle, and the change in conductivity was recorded. This experiment was performed for five succeeding cycles comprising a total of 180 s. The reversibility of the sensor was described in terms of % recovery of its initial conductivity (i.e., conductivity at zero time) after completion of the fifth cycle. For the PPy sensor at 0.5 vol %, there was a regular decrease in the conductivity after each cycle. Therefore, conductivity could not return to its original value (Figure 11). The reversibility of PPy at 0.5 vol % was found to be 72.4% because of very slow and partial desorption of ammonia molecules. However, the PPy/CeO_2_ based sensor exhibited much higher reversibility than PPy sensor. PPy/CeO_2_ based sensor showed an excellent dynamic response of electrical conductivity in ammonia vapor and ambient air (Figure 12). The reversibility of PPy/CeO_2_ based sensor at 0.5, 0.4, 0.3, 0.2, 0.1, 0.05, 0.03, and 0.01 vol % was found to be 90.6%, 91.4%, 94.6%, 95.3%, 96.1%, 98.2%, 98.9%, and 99.3%, respectively. The excellent reversibility of the PPy/CeO_2_ based sensor may be attributed to the quick and nearly complete desorption of ammonia molecules from its surface.

#### 3.6.3. Selectivity

**Figure 13:**
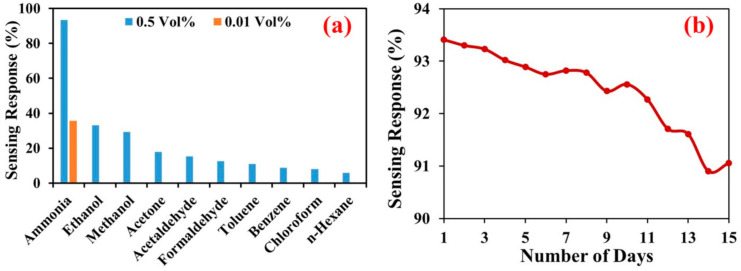
(**a**) Selectivity of PPy/CeO_2_ based sensor at 0.5 vol % ammonia concentrations against different VOCs and (**b**) stability of PPy/CeO_2_ sensor.

To determine the selectivity of the PPy/CeO_2_ based sensor towards ammonia vapor at 0.5 vol % and 0.01 vol %, its sensing response was also calculated in the presence of several other volatile organic compounds (VOCs). The % sensing response was calculated at 0.5 vol % concentration of ethanol, methanol, acetone, acetaldehyde, formaldehyde. Whereas toluene, benzene, chloroform, and *n*-hexane were utilized as received in the experiment. The % sensing response was found to 93.4%, 33.1%, 29.3%, 17.9%, 15.4%, 12.6%, 10.5%, 8.8%, 8.1%, and 5.8% towards ammonia, ethanol, methanol, acetone, acetaldehyde, formaldehyde, toluene, benzene, chloroform, and *n*-hexane, respectively (Figure 13a). Whereas, at 0.01 vol %, the sensor only responded towards ammonia vapors which showed that sensor was exclusively selective for ammonia (Figure 13a). Due to different electron-donating tendency as well as the rate of vaporization, all the tested VOCs here adsorbed to a different extent on the sensor-surface. Therefore, they interact differently with charge carriers and hence showed varying sensing response. Among all VOCs here, ammonia has the greatest electron-donating tendency along with a very high evaporation rate. Therefore, it neutralizes a large number of charge carriers which significantly reduce charge density and ultimately triggering the highest decrease in conductivity as compared to other VOCs. Thus, the PPy/CeO_2_ based sensor displayed outstanding selectivity to ammonia vapor as compared to different VOCs tested.

#### 3.6.4. Stability

The stability of PPy/CeO_2_ based sensor is evaluated by recording its % sensing response for 15 successive days. The % sensing response was calculated for successive 15 days at 0.5 vol % and found to 93.40%, 93.30%, 93.23%, 93.02%, 92.89%, 92.75%, 92.82%, 92.78%, 92.43%, 92.56%, 92.27%, 91.71%, 91.61%, 90.90%, and 91.06%, respectively (Figure 13b). Thus, after 15 days, its sensing response was decreased from 93.40% to 91.06% which indicate outstanding stability.

#### 3.6.5. Sensing Mechanism

**Figure 14:**
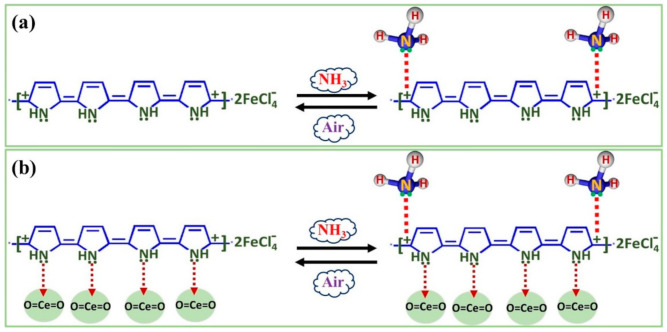
Proposed ammonia vapor sensing mechanism is showing the electronic interaction of ammonia molecules with the charge carriers of: (**a**) PPy and (**b**) PPy/CeO_2_ based sensor pellets.

The vapor/gas sensing mechanism of conducting-polymer-based sensors depend on the rate of adsorption–desorption of analyte molecules on the sensor-surface [50,51]. The adsorbed vapor/gas molecules, then interact electronically with the charge carriers. Consequently, the sensor’s electrical conductivity/resistance changes depending upon the type of vapor/gas. The electron donor species (i.e., reducing gas/vapor), for example, ammonia, reduces the charge carrier density, which causes a decrease in conductivity. When the sensor is detached from the vapor/gas’ atmosphere and exposed in air, the analyte molecules start to desorb, and interaction of analyte molecules with charge carriers decreases. Thus, the sensor’s electrical conductivity starts to increase with the increasing rate of desorption [40,41,42,43,44,50,51]. The sensor’s response/recovery time significantly depend on the rate of adsorption and desorption. The greater the sensor’s surface area, greater will be the number of active sites and hence greater will be the rate of adsorption. Therefore, the improved performance of the PPy/CeO_2_ based sensor may be related to the greater surface area provided by CeO_2_ NPs, which enabled a larger number of active sites for the adsorption of ammonia molecules (Figure 14).

The number of ammonia molecules increases on increasing concentration, which ultimately interact with a much greater number of charge carriers and reduced the density of charge carriers much faster than at lower concentrations. Hence, at higher concentrations, the % sensing response was greater along with a shorter response time. However, recovery time at higher concentrations are larger than those at lower concentration because, at higher concentrations, a large number of ammonia molecules need to be desorbed, which take a longer time (Table 2). The greater % sensing response and shorter response/recovery time of sensors based on PPy/CeO_2_ than PPy sensor also revealed the possibility of more rapid adsorption and desorption process of ammonia molecules on PPy/CeO_2_ sensor pellet as compared to PPy.

A comparison of sensing parameters of this PPy/CeO_2_ sensor with the previously published ammonia sensing studies on PPy based sensors are given in Table 3.

## 4. Conclusions

We report the preparation and characterization of polypyrrole (PPy) and polypyrrole/cerium oxide (PPy/CeO_2_) nanocomposites for the fabrication of a promising pellet-shaped ammonia vapor sensor. DC electrical conductivities of PPy and PPy/CeO_2_ were found to be 0.152 Scm^−1^ and 1.295 Scm^−1^, respectively at room temperature. PPy/CeO_2_ nanocomposite showed much greater conductivity retention properties than PPy under both the isothermal and cyclic ageing conditions. PPy/CeO_2_ sensor exhibit much higher sensing performance than pristine PPy sensor at 0.5 vol % ammonia concentration in terms of % sensing response, response/recovery time and reversibility. The sensing performance of the PPy/CeO_2_ sensor at room temperature was also studied at 0.4. 0.3, 0.2, 0.1, 0.05, 0.03, and 0.01 vol % ammonia concentration in terms of % sensing response, response/recovery time, reversibility, selectivity and stability. The response and recovery time for the PPy sensor at 0.5 vol % ammonia concentration was found to be about 80 s/150 s, respectively. However, response/recovery time for PPy/CeO_2_ based sensor 0.5 vol % ammonia concentration was found to be about 40 s/60 s, respectively. The % sensing response of PPy/CeO_2_ sensor was also calculated at 0.4, 0.3, 0.2, 0.1, 0.05, 0.03, and 0.01 vol % ammonia concentrations and found to be 76.2%, 68.8%, 61.4%, 57.5%, 48.2%, 41.6%, and 35.6%, respectively. The response and recovery time of the PPy/CeO_2_ sensor varies significantly as ammonia concentration changes from 0.01 vol % to 0.5 vol %. The response/recovery time was found to be 45 s/47 s, 52 s/40 s, 56 s/38 s, 59 s/30 s, 65 s/26 s, 69 s/23 s, and 75 s/20 s at 0.4, 0.3, 0.2, 0.1, 0.05, 0.03, and 0.01 vol % ammonia concentrations, respectively. The reversibility of PPy/CeO_2_ sensor at 0.5, 0.4, 0.3, 0.2, 0.1, 0.05, 0.03, and 0.01 vol % was found to be 90.6%, 91.4%, 94.6%, 95.3%, 96.1%, 98.2%, 98.9%, and 99.3%, respectively along with exceptional selectivity towards ammonia vapor.

## Figures and Tables

**Table 1:** Characteristic peaks of PPy and PPy/CeO_2_.

S. No.	Materials	N-H (cm^−1^)	C=C (cm^−1^)	C-C (cm^1^)	C=N (cm^1^)	C-N (cm^1^)	=C-H (cm^1^)	C=N^+^-C (cm^−1^)	C-H (cm^1^)
1.	PPy	3430.8	1550.8	1459.9	1307.1	1187.3	1044.8	922.5	791.9
2.	PPy/CeO_2_	3427.7	1542.6	1448.1	1305.6	1171.3	1037.2	906.5	784.4

**Table 2:** Summarized results of PPy/CeO_2_ based sensor pellet towards different concentrations of ammonia vapor.

S. No.	Ammonia-Concentration (vol %)	Sensing Response (%)	Response Time (s)	Recovery Time (s)	Reversibility (%)
1.	0.5	93.4	40	60	90.6
2.	0.4	76.2	45	47	91.4
3.	0.3	68.8	52	40	94.6
4.	0.2	61.4	56	38	95.3
5.	0.1	57.5	59	30	96.1
6.	0.05	48.2	65	26	98.2
7.	0.03	41.6	69	23	98.9
8.	0.01	35.6	75	20	99.3

**Table 3:** Comparison of present study with the other ammonia sensors based on PPy.

S. No.	Material Used	Method of Preparation	Response (%)	Response Time	Recovery Time	Reference
1.	PPy/RGO	Chemical oxidative	50 at 10 ppm	200 s	90 s	[1]
2.	SnO_2_/ZnO/PPy	Electro-spinning method	0.68 at 30 ppm	67 s	106 s	[2]
3.	PPy thin films	Solution casting	12 at 25 ppm	90 s	10 min	[3]
4.	PPy/Au	Chemical oxidative	50 at 300 ppm	40 s	80 s	[4]
5.	PPy/Zn_2_SnO_4_	Layer-by-layer self-assembly	82.1 at 100 ppm	35 s	26 s	[5]
6.	PPy/MoS_2_	Chemical oxidative	-	60 s	50 s	[17]
7.	PPy/CeO_2_	Chemical oxidative	93.4 at 0.5 vol % and 35.6 at 0.01 vol %	40 s at 0.5 vol % and 75 s at 0.01 vol %	60 s at 0.5 vol % and 19 s at 0.01	This study

## Data Availability

The data presented in this study are available on request from the corresponding author.
